# Building advocacy into research

**DOI:** 10.1242/dmm.050646

**Published:** 2023-12-18

**Authors:** James F. Amatruda

**Affiliations:** Cancer and Blood Disease Institute, Children's Hospital Los Angeles, 4650 Sunset Boulevard, MS 57, Los Angeles, CA 90027, USA

**Figure:**
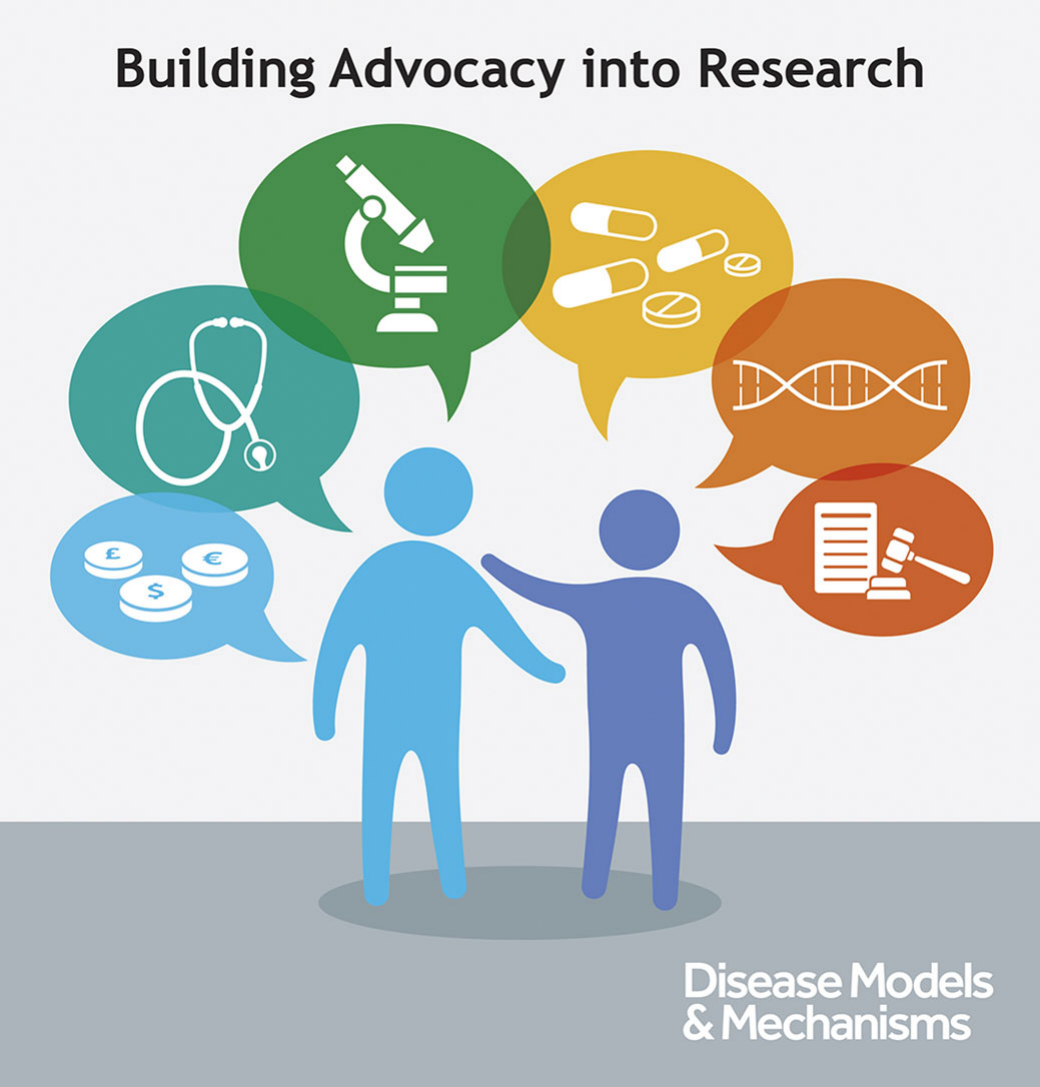
This image is by neilsmithillustration.co.uk and published under the CC-BY 4.0 license for this article.

This issue of Disease Models & Mechanisms introduces a new series, ‘Building Advocacy into Research’, which is an initiative aimed at fostering communication and mutual support between the research community and the patients, families and advocates impacted by disease.

Fifteen years ago, DMM was founded on the idea that ‘application of fundamental discoveries from (model) organisms will accelerate our understanding, diagnosis and treatment of human diseases’, and with a goal to ‘foster a connection between the model organism scientists and the clinicians’ (Freeman and St Johnston, 2008). This vision was affirmed and expanded by Editor-in-Chief Liz Patton in 2021, recognizing that the disease biology field needs to extend well beyond model organisms and incorporate human cell and tissues, veterinary animals, evo-devo approaches, advanced bioengineering and *in vitro* systems. These systems truly enable researchers to define mechanisms of disease with ‘science that pushes the boundaries of disease biology and makes strong links with clinical science’ (Patton, 2021).

In discussions amongst the Editors, it seemed to us that a missing component of this vision was the perspective of patients impacted by disease and the advocates dedicated to driving progress and improving outcomes for patients. In the clinical arena, the act of listening to the patient's voice has increasingly been recognized as an essential component of effective care (Dorr Goold and Lipkin, 1999; Larkin, 2020) and of the education of clinical caregivers (Stewart et al., 1986; Ranjan et al., 2015). In the past few decades, this concept has grown to include the vital role of patient and advocate perspective in clinical research (Marsden et al., 2004; Chad et al., 2013; Cobb et al., 2015; Bhardwaj et al., 2019). Involving patients and advocates in the design of clinical research can help facilitate enrollment and promote the important goal of equity and inclusion of a diverse spectrum of patients in clinical trials (Pate et al., 2021; Halley et al., 2023). Patient voices are also critical for helping to set research priorities, address ethical concerns, and bring attention to issues of toxicity and other burdens faced by patients participating in clinical research (Cohen et al., 2007; Bhardwaj et al., 2019; Martinez, 2019).

It is important to note that the roles of patients and advocates in research, both basic and clinical, go well beyond simply increasing the numbers of participants in a study, or raising issues of equity, ethics or logistical barriers. Patient advocacy groups have emerged as a potent force in biomedical research because, in many cases, these groups have had to build networks of cooperation and mutual support from the ground up. Especially in the case of rare diseases, patients and families can face the frustration of delayed diagnoses, fragmented care among different specialists, and a lack of therapeutic options and clinical trials. For these reasons, patients and advocates have built strong communities by sharing their experiences and by helping to bring about better clinical options for patients affected by particular conditions (House et al., 2019; Huml et al., 2021; Patterson et al., 2023).

As a perhaps natural consequence of the sense of community and urgency that characterizes many patient advocacy groups, these organizations, both large and small, have been at the forefront of galvanizing progress, by educating clinicians on the real-world problems faced by patients confronted with different diseases, and catalysing fundraising efforts to directly support basic and clinical research. This is especially important in the case of rare diseases for which, historically, commercial biopharmaceutical companies may have been disincentivized from investing in research (Wainstock and Katz, 2023; Yoo, 2023). That paradigm may, however, be changing, thanks to the effective role of patients and advocates in engaging with legislators and other policymakers to improve funding for research, patient access to new therapies and the regulatory environment for research and approval of new drugs (Dharssi et al., 2017; Paradise, 2018; Folkers et al., 2019).

How can this perspective of patient and advocate inclusion extend to basic and translational pre-clinical research? What are the benefits, to patients/advocates and to researchers, of increased engagement? When it comes specifically to the involvement of patients and advocates in basic and translational pre-clinical work, there are additional opportunities, and challenges, to consider. In many cases, patients are willing to generously participate directly in research by allowing use of their biospecimens and clinical/demographic data, which can help to both drive basic discovery efforts and ensure the rigor and representativeness of disease models by providing a ‘gold standard’ of human tissue material. Especially for rare diseases, patient advocacy groups can help directly connect patient communities to researchers conducting disease-focused work. For researchers, this type of participation by patients entails certain obligations, both to carefully steward precious research materials and to address very important questions of equity, privacy and genetic discrimination (Suter, 2004; Korobkin and Rajkumar, 2008; Rice et al., 2023).

Equity of access to research initiatives is of paramount importance. As patients from under-resourced communities and demographic groups may not have as much opportunity to raise funds or organize into advocacy groups (Halley et al., 2023), researchers may need to make active efforts to engage with these communities in creative ways. The issue of genetic privacy is heightened by the increasing sophistication of genomic technologies, where the widespread use of whole-genome assays for characterization of patient-derived tissues may generate genetic signatures that can be traced back to a specific donor, even for deidentified samples (Santalo and Berdasco, 2022). Efforts to minimize this risk may conflict with the valuable goal of promoting the sharing of data, to enable replication of results and promote further research and discovery. In striking the balance between risk and benefits for omics research, it is essential for researchers to engage with patients and advocates to try to address their concerns.

Just how to go about these essential conversations can be a challenge, as the technical aspects of genome characterization, data sharing and other aspects of modern disease research can be difficult to convey clearly in layperson's terms. Indeed, patients have identified ‘differences in knowledge and research experience’ as a possible impediment to communication and collaboration (Fox et al., 2021). One possible solution to this challenge is to increase the involvement of advocates as members of the research team, with ongoing, long-term interactions providing an opportunity for advocates to become conversant with the goals and procedures of pre-clinical research, and empowering them to serve as educators within disease advocacy communities. These interactions can take many forms: patients and advocates may participate in reviewing research proposals or in writing proposal guidelines – for example, prioritising specific topics or requiring lay summaries as part of the grant application. To become familiar with new research initiatives, patients and advocates might receive hands-on training and participate in research activities and research presentations, from lab meetings to public seminars to national and international conferences.

As with other aspects of patient engagement, it will be important to be cognizant that this participation is not without costs, in terms of time off from work, travel, conference registration fees and other expenses. Where possible, funding bodies could provide finances to help defray these expenses, which would be a valuable way to support advocacy efforts and the long-term goal of improving outcomes for patients.

Finally, perhaps the most important and most effective way for disease researchers to engage with patients and advocates is to start by listening. Patients and advocates have much to teach us as researchers. Their passion, commitment and sense of urgency can be a powerful motivator and source of inspiration for research groups, as well as providing the unique perspectives discussed above. In the spirit of listening, the ‘Building Advocacy into Research’ series will feature interview articles, titled ‘The Patient's Voice’, with patients and advocates across a range of disease types. We are launching this series with interviews with Anne-Marie Baird from Lung Cancer Europe, Marie Ojiambo from the Sickle Strong Initiative and Nicole Polinski from the Michael J. Fox Foundation. It is our hope that the series will provide patients and researchers alike a set of roadmaps for how to build strong, ongoing partnerships, supporting the highest-quality research for the benefit of all patients affected by disease.
